# Microbial Eukaryote Diversity and Activity in the Water Column of the South China Sea Based on DNA and RNA High Throughput Sequencing

**DOI:** 10.3389/fmicb.2017.01121

**Published:** 2017-06-14

**Authors:** Dapeng Xu, Ran Li, Chen Hu, Ping Sun, Nianzhi Jiao, Alan Warren

**Affiliations:** ^1^State Key Laboratory of Marine Environmental Science, Institute of Marine Microbes and Ecospheres, Xiamen UniversityXiamen, China; ^2^Key Laboratory of the Ministry of Education for Coastal and Wetland Ecosystem, College of the Environment and Ecology, Xiamen UniversityXiamen, China; ^3^Department of Life Sciences, Natural History MuseumLondon, United Kingdom

**Keywords:** biological pump, deep sea, metabolic activity, protist, RNA/DNA ratio

## Abstract

To study the diversity and metabolic activity of microbial eukaryotes in the water column of the South China Sea, genomic DNA and RNA were co-extracted from samples collected down to bathyal depth at two sites. V9 regions of both SSU rRNA gene and its transcript (cDNA) were amplified and sequenced using high throughput sequencing. Our study revealed: (1) DNA and RNA datasets showed significant differences in microbial eukaryote community composition, with the variability between the two datasets for the same sample exceeding that between samples within each dataset, indicating that nucleic acid source overrode environmental factors in determining the composition of microeukaryotes; (2) despite the differences in community composition between the two datasets, both DNA and RNA revealed similar depth-related distribution patterns of microbial eukaryotes; (3) using the ratio of RNA: DNA as a proxy of relative metabolic activity, a depth-related pattern was found for the relative metabolic activity of some but not all groups of microbial eukaryotes, with the highest activity for the groups with depth-related pattern usually found in the middle water layers; and (4) the presence of live and active photoautotrophic microbial eukaryotes in the deep ocean was confirmed, indicating that they play an important role in controlling the deep-sea organic carbon pool. Overall, our study sheds light on the diversity and activity of microbial eukaryotes in the water column of a tropical oligotrophic ocean and their potential contributions in the downward transportation of organic material from the surface ocean to the deep via the biological pump.

## Introduction

Single-celled eukaryotes (protists) comprise a wide range of morphologically, genetically, and functionally diverse lineages that act as primary producers, consumers, decomposers, parasites, and/or trophic links in microbial food webs. They thus play key roles in global biogeochemical cycles of numerous elements including carbon, nitrogen, and silica (Azam et al., 1983; Jiao et al., 2010; Caron et al., 2012; Worden et al., 2015). Studies on the taxonomy, diversity, and phylogeny of protists based on conventional methods, e.g., observations by microscopy of specimens *in vivo* or following fixation and staining, have been carried out for centuries and have laid a strong foundation for our understanding of their biology and diversity (Whittaker, 1969). In recent decades, the application of culture-independent methods (especially DNA-based sequencing) to natural samples collected from a wide range of aquatic and terrestrial ecosystems has played a central role in the discovery of novel microbial eukaryote lineages (López-García et al., 2001a,b; Stoeck et al., 2003; Massana et al., 2004; Not et al., 2007; Dunthorn et al., 2014) as well as revealing their patterns of distribution (Rodriguez-Martinez et al., 2013; Logares et al., 2014; de Vargas et al., 2015; Grattepanche et al., 2016; Santoferrara et al., 2016; Sun et al., 2016).

Environmental DNA samples usually consist of nucleic acids derived from living, dormant (e.g., cysts) and dead microbial cells (Josephson et al., 1993), as well as extracellular free DNA (Dell'Anno and Danovaro, 2005). Recent studies have shown the importance of differentiating the active from the total communities (Jones and Lennon, 2010; Campbell et al., 2011). Moreover, the SSU rRNA gene copy number varies from one to thousands in single eukaryotic genomes (Zhu et al., 2005; Godhe et al., 2008; Gong et al., 2013) making it difficult to directly link read numbers to abundances of individual organisms. Compared with DNA, extracellular RNA molecules are much less stable and survive for much shorter time periods (Karl and Bailiff, 1989). By targeting RNA, the problems associated with interference by nucleic acids from inactive/dead cells, extracellular nucleic acids, and differences in rRNA gene copy number, can be avoided. Furthermore, the community diversity revealed by RNA responds more sensitively to environmental conditions than that revealed by DNA (Charvet et al., 2014). There are thus several advantages of including both DNA and RNA when analyzing environmental nucleic acids. This approach has been proven to be effective for prokaryotes (Mills et al., 2005; Moeseneder et al., 2005; Gentile et al., 2006), but has only recently been applied to protistan community surveys (Stoeck et al., 2007; Not et al., 2009; Orsi et al., 2013; Logares et al., 2014; de Vargas et al., 2015). In addition, only a few studies have applied RNA: DNA ratios to infer the metabolic activity of microbial eukaryotes assemblages, and these were either restricted to protist communities above the euphotic/disphotic zones (Charvet et al., 2014; Logares et al., 2014; Massana et al., 2015; Hu et al., 2016) or focused on communities in shallow coastal water sediments (Massana et al., 2015). The activity of microbial eukaryote assemblages at bathyal depths in oligotrophic waters remains largely unknown.

In this study, we investigate the diversity and activity of microbial eukaryotes in the water column, from the surface to the bathyal zone, of the South China Sea which is one of the world's largest marginal seas and represents typical tropical oligotrophic waters (Gong et al., 1992). Microbial eukaryotes were sampled at discrete depths down the water column at two separate locations. Small subunit (SSU) rRNA gene and cDNA (reverse transcribed from extracted RNA, hereafter referred to as RNA) tags were sequenced by high-throughput sequencing based on total RNA and DNA co-extracts of 32 samples. This study aimed to address the following questions: (I) what is the community structure of active microbial eukaryotes in the water column of the South China Sea? (II) do DNA and RNA reveal similar alpha and beta diversities of microbial eukaryotes and to what extent do they differ? (III) what is the relative metabolic activity of major eukaryotic assemblages in the water column at the two locations?

## Materials and methods

### Sample collection

Sampling was conducted on board of R/V Dongfanghong 2 in summer of 2014. Two sites, H7 (116°E, 14'N) and F3 (118°E, 16'N) located in the mid-region of the South China Sea, were sampled on 11 and 13 July 2014, respectively (Figure S1). Seawater was collected from eight discrete depths from the surface to the bathyal zone at both sites: F3 (25, 75, 200, 300, 500, 1,000, 1,500, and 2,000 m) and H7 (5, 25, 75, 200, 500, 1,000, 1,500, and 3,900 m).

### DNA/RNA co-extraction, PCR amplification, and sequencing

Total DNA and RNA were extracted simultaneously from each cryopreserved filter membrane using AllPrep DNA/RNA kit (Qiagen, USA) based on an initial mechanical cell disruption employing bead beating (Stoeck et al., 2007) followed by chemical disruption using lysis buffer provided in the kit. Total DNA and RNA were quantified using a Nanodrop ND-2000 Spectrophotometer (Labtech International) and the quality was assessed by gel electrophoresis. After treatment with DNase (Qiagen, Hilden, Germany) to remove the carry-over genomic DNA, RNA was then transcribed into cDNA using QuantiTect® Reverse Transcription kit (Qiagen, USA) according to the manufacturer's instructions. The universal forward and reverse primers, 1389F (5′-TTG TAC ACA CCG CCC-3′) and 1510R (5′-CCT TCY GCA GGT TCA CCT AC-3′), were used to amplify the hyper-variable loop V9 of the DNA and RNA (Amaral-Zettler et al., 2009). Six individual PCR reactions for each sample were run and later pooled. The resulting PCR amplicons (ca. 130 bp) were excised from the gel using Gel extraction kit (Promega, Shanghai, China). Bridge amplification and paired-end sequencing of the amplicons were performed with an Illumina MiSeq sequencer by a commercial sequencing company. All sequence data generated in this study have been submitted to the NCBI Sequence Read Archive and are accessible under the accession number SRP104547.

### Data processing

Overlapping reads were merged using the program FLASH with default parameters (Magoc and Salzberg, 2011). Barcoded datasets were de-multiplexed and filtered to remove low quality sequences using QIIME (Caporaso et al., 2010). Chimeras were detected and removed with UCHIME (Edgar et al., 2011) using both *de novo* and reference-based chimera searches against PR2 (de Vargas et al., 2015). For each sample, clean reads were dereplicated. Reads present as a single copy (singleton) were also removed. Reads were then clustered into Operational Taxonomic Units (OTUs) at 95% sequence similarity using UPARSE (Edgar, 2013). The taxonomy assignment of OTUs was achieved by UCLUST against the PR2 database to detect and remove taxa that are not affiliated with eukaryotes (e.g., Bacteria, Archaea, and plastidial sequences) (de Vargas et al., 2015). The OTUs affiliated with Metazoa or Unassigned were removed from the dataset before downstream analysis because the former would distort the relative abundance of DNA sequences of microbial eukaryotes and the latter are taxonomically uninformative. To normalize sampling effort, OTU counts were rarefied at a uniform sequencing depth based on the lowest sequence count (20,204 sequences) from the 75 m depth of site F3 retrieved from RNA samples (F3.75R). Following removal of low quality reads, potential chimeras, reads that were not assigned as eukaryotes (including Unassigned) and singletons, there were 2,309,424 sequences representing both protistan and metazoan taxa, ranging 22,481 to 174,306 reads per sample (Table 1). After removing sequences affiliated with Metazoa, there was a total of 2,067,712 microbial eukaryote sequences, ranging 20,204 to 169,189 sequences per sample. Using a 95% sequence similarity threshold, sequences were further clustered into 2,799 protistan OTUs. The number of protistan OTUs ranged 918–1,504 per sample (Table 1). Alpha diversity estimations (Chao1, Shannon, ACE, Inverse of Simpson, and Phylogenetic Diversity) were calculated using QIIME (Caporaso et al., 2010).

**Table 1:** Diversity parameters of South China Sea samples.

**Sample**	Clean reads^*^	**Metazoa reads (%)**	**Clean reads (metazoa reads excluded)**	**Metazoan OTUs**	**OTU_0.05_ (metazoan OTUs excluded)**	OTU_0.05_^**^	Chao1^**^	ACE^**^	Shannon^**^	Simpson ^**^	PD^**^
F3.25D	54,581	13.0	47,484	64	1,371	1,065	1,418	1,417	6.34	0.015	163.15
F3.75D	34,495	18.5	28,117	67	1,125	1,031	1,346	1,331	7.25	0.039	159.29
F3.200D	35,586	16.2	29,805	50	1,262	1,097	1,491	1,456	7.09	0.031	164.15
F3.300D	77,840	5.0	73,968	75	1,459	952	1,429	1,398	5.54	0.012	150.66
F3.500D	99,944	9.1	90,828	76	1,385	836	1,201	1,218	5.26	0.013	129.84
F3.1KD	150,687	4.7	143,668	70	1,323	633	918	968	4.47	0.013	111.00
F3.15KD	148,897	5.1	141,365	78	1,268	599	914	1,022	3.40	0.009	110.85
F3.2KD	174,306	2.9	169,189	67	1,381	669	1,083	1,151	4.16	0.009	120.26
F3.25R	29,060	8.7	26,522	49	1,172	1,092	1,380	1,401	8.06	0.105	173.86
F3.75R	22,481	10.1	20,204	43	918	918	1,239	1,196	7.67	0.093	148.53
F3.200R	24,836	12.3	21,772	39	1,032	1,007	1,288	1,246	7.12	0.022	157.56
F3.300R	31,288	4.2	29,968	45	1,147	1,011	1,413	1,337	7.00	0.031	158.61
F3.500R	40,374	7.5	37,332	50	1,129	942	1,215	1,213	7.03	0.042	154.67
F3.1KR	52,331	4.8	49,802	52	1,161	867	1,120	1,118	5.92	0.016	141.83
F3.15KR	67,120	3.6	64,716	53	1,026	665	1,037	1,081	3.91	0.005	119.70
F3.2KR	63,444	2.8	61,696	53	1,111	771	1,048	1,100	5.89	0.029	134.28
H7.5D	38,986	41.5	22,815	65	1,119	1,082	1,426	1,421	7.62	0.052	162.61
H7.25D	35,680	32.7	24,013	60	1,320	1,272	1,702	1,647	7.88	0.052	180.16
H7.75D	52,050	11.1	46,285	62	1,451	1,098	1,526	1,497	6.84	0.028	166.21
H7.200D	70,027	17.9	57,510	85	1,504	1,078	1,509	1,486	6.49	0.021	164.96
H7.500D	31,488	11.2	27,946	59	1,155	1,051	1,420	1,450	6.80	0.030	161.60
H7.1KD	122,774	23.4	94,077	83	1,398	836	1,269	1,335	5.11	0.012	133.54
H7.15KD	134,465	12.1	118,161	80	1,330	664	999	1,065	4.26	0.011	115.79
H7.39KD	118,192	7.1	109,746	94	1,498	810	1,329	1,295	5.13	0.013	140.79
H7.5R	40,821	21.8	31,924	66	1,003	901	1,230	1,199	7.48	0.081	147.53
H7.25R	55,457	42.1	32,131	70	1,155	1,027	1,429	1,404	7.48	0.061	162.22
H7.75R	56,374	11.6	49,842	68	1,300	994	1,352	1,377	6.81	0.029	161.07
H7.200R	50,270	17.2	41,628	80	1,238	985	1,398	1,377	6.42	0.022	160.56
H7.500R	37,634	5.0	35,734	61	965	789	1,194	1,134	6.34	0.029	133.99
H7.1KR	114,111	4.9	108,558	66	1,187	679	1,021	1,087	4.93	0.019	121.41
H7.15KR	133,136	7.3	123,380	67	1,127	579	976	1,016	4.02	0.010	111.64

*OTU_0.05_, Operational taxonomic unit at 95% SSU rRNA V9 gene sequence identity*.

* Reads affiliated with protists only;

** *Standardized numbers based on subsampling of 22,875 sequences without replacement*.

An OTU table generated in QIIME was used to study the similarities among microbial eukaryote communities of the 32 samples using the Bray-Curtis coefficient within PRIMER v.6 (Clarke and Warwick, 2001). OTU abundances were normalized to relative abundances within each library using a square-root transformation to prevent a few well-represented OTUs from driving the similarity analysis (Clarke and Warwick, 2001). ANOSIM was conducted to establish the significance of dendrogram nodes resulting from cluster analysis. To check the robustness of dissimilarity among communities, beta diversity was calculated using the unweighted Unifrac metric, which compares samples based on the phylogenetic relatedness of OTUs in a community without taking into account relative OTU abundance (Lozupone and Knight, 2005). Visualization of community dissimilarity based on the unweighted Unifrac metric was made using a two-dimensional Principal Coordinate Analysis (PCoA). Simple Mantel tests were conducted in R with the Vegan package to assess correlations between environmental variables and community variability (Legendre and Legendre, 1998).

### Relative metabolic activity of major microbial eukaryote assemblages

To reveal the relative metabolic activity of major microbial eukaryote assemblages for samples collected at each water depth, OTUs occurring only in the DNA or only in the RNA dataset were not included in the analysis (Hu et al., 2016). RNA: DNA ratios for each OTU were calculated, then average ratios for each major microbial eukaryote group were used as a proxy for relative activity of the group (Hu et al., 2016). To reveal the differences in RNA: DNA ratios of different groups of microbial eukaryotes, OTUs from the same group and water layers (shallow: 5, 25, and 75 m; middle: 200, 300, and 500 m; deep: 1,000, 1,500, 2,000, and 3,900 m) were pooled and the differences were evaluated using analysis of variance (ANOVA) (*P* < 0.05).

## Results

### Sampling sites and environmental factors

Two sites were chosen to represent the central part of the South China Sea (Figure S1). The physical properties of the oceanic waters at these two sites exhibited similar environmental conditions at each of the depths sampled (Figure S2). A well-defined pycnocline was found with the steepest gradients in salinity (33.1 to 36.4 psu) and temperature (ca 30.2 to 2.4°C) between 20 and 100 m (Figure S2). The bacterial abundance decreased with increasing depth, from ca. 7.4 × 10^5^ to 8.4 × 10^4^ cells ml^−1^ between 25 and 2,000 m water depths at site F3 and from ca. 6.7 × 10^5^ to 4.3 × 10^4^ cells ml^−1^ between 5 and 3,900 m at site H7.

### Alpha and beta diversity of microbial eukaryotes in the water column of the South China sea

Metazoa represented approximately 2.9–42.1% of total eukaryote reads in each sample. The proportions of metazoan sequences generally decreased with increasing water depth at both sites (Table 1). For the same sample for which DNA and RNA were co-extracted and sequenced, the proportions of metazoan sequences were smaller in RNA than in DNA amplicons in the majority of the samples. One exception was the sample collected at 25 m at site H7, for which the proportion of metazoan sequences was higher in the RNA extract (ca. 42.1%) than in the DNA extract (32.7%). Other exceptions were the samples collected at 1,000 m at site F3 and 75 m at site H7, where the proportions of metazoan sequences were only slightly higher in the RNA than in the DNA extracts (4.8 vs. 4.7% and 11.6 vs. 11.1%, respectively).

To calculate alpha diversity and evaluate differences in microbial eukaryote community composition between samples, OTU counts were rarefied at a uniform sequencing depth based on the lowest sequence count from sample F3.75R, i.e., RNA extract from sample collected at 75 m at site F3 (*n* = 20,204 sequences). As a general trend, the richness decreased with increasing depth but increased again at the greatest depths sampled (2,000 m at F3 and 3,900 m at H7, respectively). In the RNA extracts, the highest richness of microbial eukaryotes was at 25 m (1,092 OTUs) and the lowest was at 1,500 m (1,037 OTUs). At site H7, the highest richness was at 25 m and the lowest was at 1,500 m, both in DNA (1,272 and 664 OTUs, respectively) and RNA (1,027 and 579 OTUs, respectively) extracts. Site F3 showed a similar trend with the lowest richness at 2,500 m, however the highest was at 200 m (1,097 OTUs) in the DNA extracts and at 25 m (1,092 OTUs) in RNA extracts. The four diversity estimators, i.e., Chao1, ACE, Shannon, and Simpson, showed the same trend (Table 1). Phylogenetic diversity (PD), which measures the total branch length connecting all OTUs in the SSU rRNA gene phylogeny, also showed the same trend (Table 1).

Four supergroups dominated the microbial eukaryote assemblages at the two sites regardless of the water depth: Alveolata, Rhizaria, Stramenopiles, and Excavata (Figure S3). In terms of OTU richness, the pattern of distribution of these supergroups was similar at the two sites retrieved either with DNA or RNA. The proportions of each supergroup did not change significantly with increasing depth. In the DNA survey, these four supergroups represented 86–90% of the total richness (Figures S3A,B). In the RNA survey, the same four supergroups dominated with comparable, but slightly smaller, contributions, i.e., 80–85% of total richness (Figures S3C,D).

**Figure 1:**
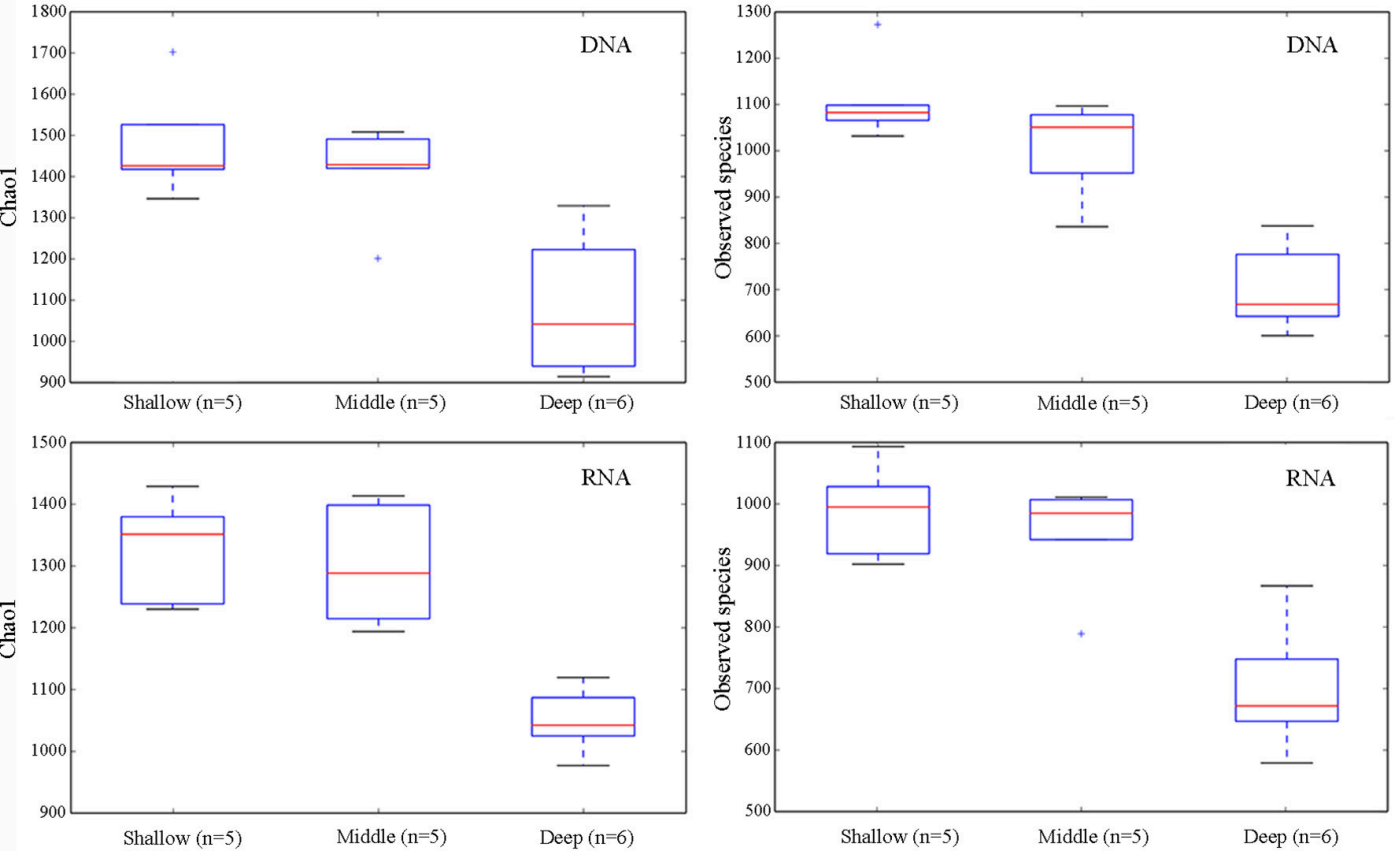
Alpha-diversity measures (Chao1 and Observed species) for the pooled samples from the two sampling sites grouped by water depths, shallow (5, 25, and 75 m), middle (200, 300, and 500 m), and deep (1,000, 1,500, 2,000, and 3,900 m), revealed by DNA and RNA, respectively. The line in each box plot indicates the median, the box delimits the 25th and 75th percentile, and the whisker is the range.

To reveal the differences of alpha-diversity along the depth gradients, the samples were categorized into three groups: shallow (5, 25, and 75 m), middle (200, 300, and 500 m), and deep (1,000, 1,500, 2,000, and 3,900 m). The nonparametric test (Monte Carlo permutations) based on the Chao1 and Observed species showed that the alpha-diversity of the shallow water group was not significantly different from the middle group in both the DNA and RNA surveys. By contrast, there were significant differences in the alpha-diversity between the shallow and the deep groups and between the middle and the deep groups (Figure 1).

**Figure 2:**
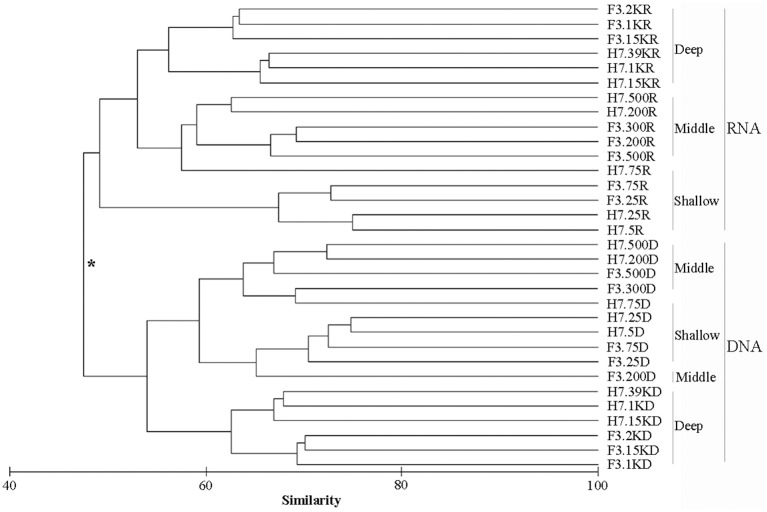
Cluster diagram of Bray-Curtis similarities calculated from square-root transformed relative OTU abundances for each sample. The asterisk at the node in the dendrogram separating DNA and RNA surveys indicates significant compositional differences between these two groups (R = 0.5506, *p* = 0.0001) determined by the ANOSIM.

The samples were clustered into two groups, first by nucleic acid sources based on the Bray-Curtis similarity, i.e., samples retrieved from DNA and samples retrieved from RNA, respectively (Figure 2). This grouping pattern was also supported by the principle component analysis of community taxonomic relatedness quantified by the Unweighted Unifrac metric (Figure S4). Statistical analyses showed the composition of the two clusters was significantly different (ANOSIM, R = 0.5506, *p* = 0.0001). Within the RNA cluster, the samples were generally grouped by water depth. Samples collected from deep waters (over 1,000 m) formed a group which then clustered with samples from the middle layers (200, 300, and 500 m) plus the sample from 75 m at site H7, and these two groups formed a sister group to the samples retrieved from shallow waters (5, 25, and 75 m). The samples within the DNA cluster were also grouped by water depth, except that samples from middle layers clustered first with those of shallow waters rather than with samples from deep layers.

### Community composition of microbial eukaryotes revealed by DNA and RNA surveys

**Figure 3:**
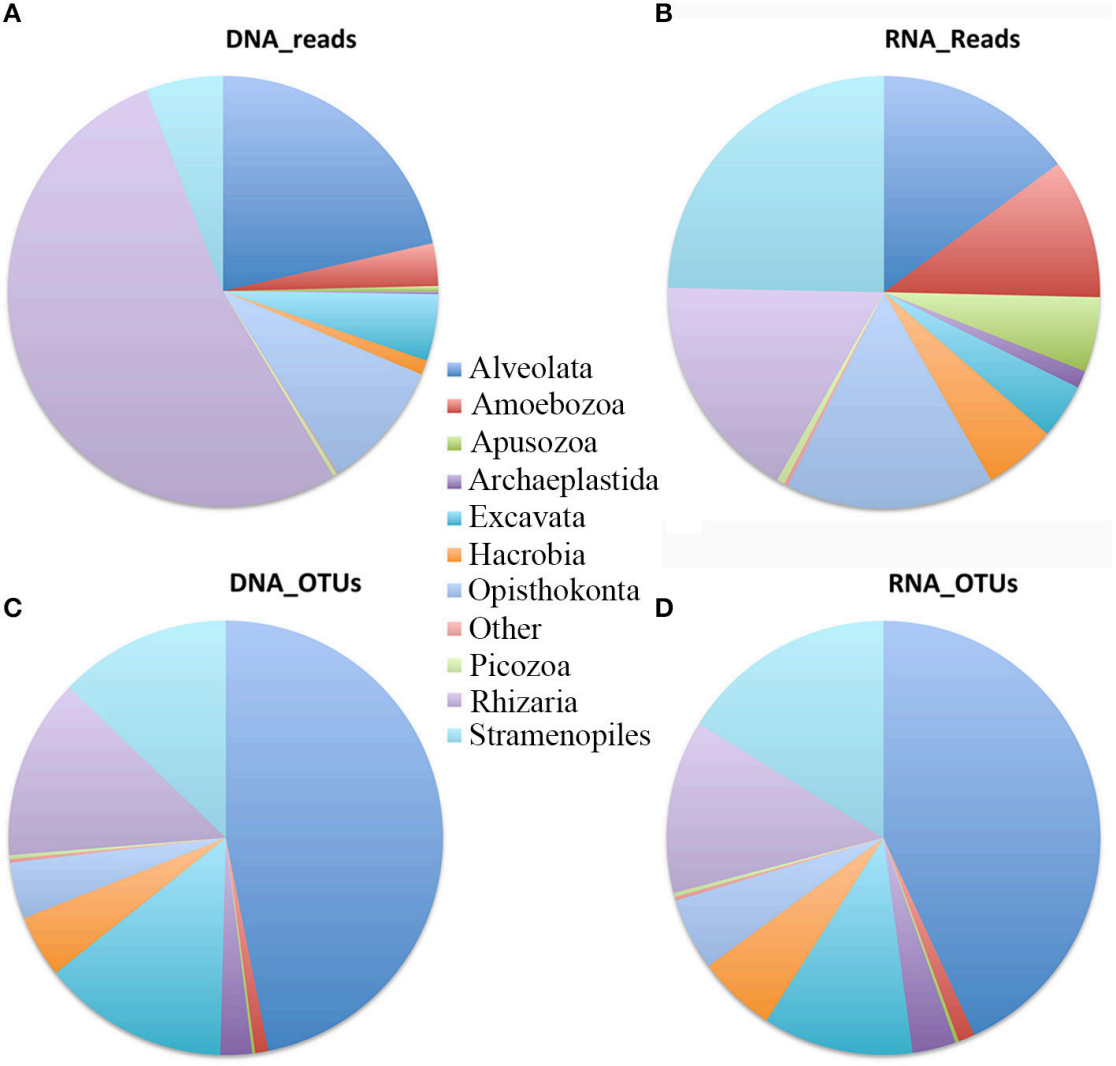
Overview of assemblages of microbial eukaryotes in the South China Sea water column at the supergroup taxonomic level. Relative sequence number in DNA survey **(A)** and RNA survey **(B)**; relative OTU number in DNA survey **(C)** and RNA survey **(D)**.

Pooling all data from different depths and sites gave the first insight of the microbial eukaryote community compositions at different sites and depths in the South China Sea revealed by DNA and RNA datasets. The reads affiliated with Rhizaria (mainly Cercozoa and Radiolaria) represented over half of the total DNA reads followed by Alveolata (21%), Opisthokonta (9.7%) (represented mainly by Fungi and Choanoflagellida), and Stramenopiles (5.8%). The other supergroups, i.e., Excavata, Amoebozoa, Apusozoa, Archaeplastida, Hacrobia, and Picozoa, collectively contributed only ca. 10.8% of total reads (Figure 3A). Compared with the DNA dataset, reads in the RNA survey were relatively evenly distributed among the different supergroups. Stramenopiles was the dominant supergroup, representing 24.7% of total microbial eukaryote reads. Rhizaria was the second most dominant with 17.1% of total reads, followed by Opisthokonta (15.6%), Alveolata (14.9%), and Amoebozoa (10.5%) (Figure 3B). However, in terms of OTU richness, DNA and RNA showed similar patterns in the three most dominant supergroups, i.e., Alveolata, Stramenopiles, and Rhizaria, accounting for 47, 12.8, and 13.4% in DNA and 43, 16.2, and 12.9% in RNA surveys, respectively. Excavata (mainly Euglenozoa and other unidentified Discoba) represented 14% of total DNA OTUs and 11% of total RNA OTUs, respectively (Figures 3C,D).

**Figure 4:**
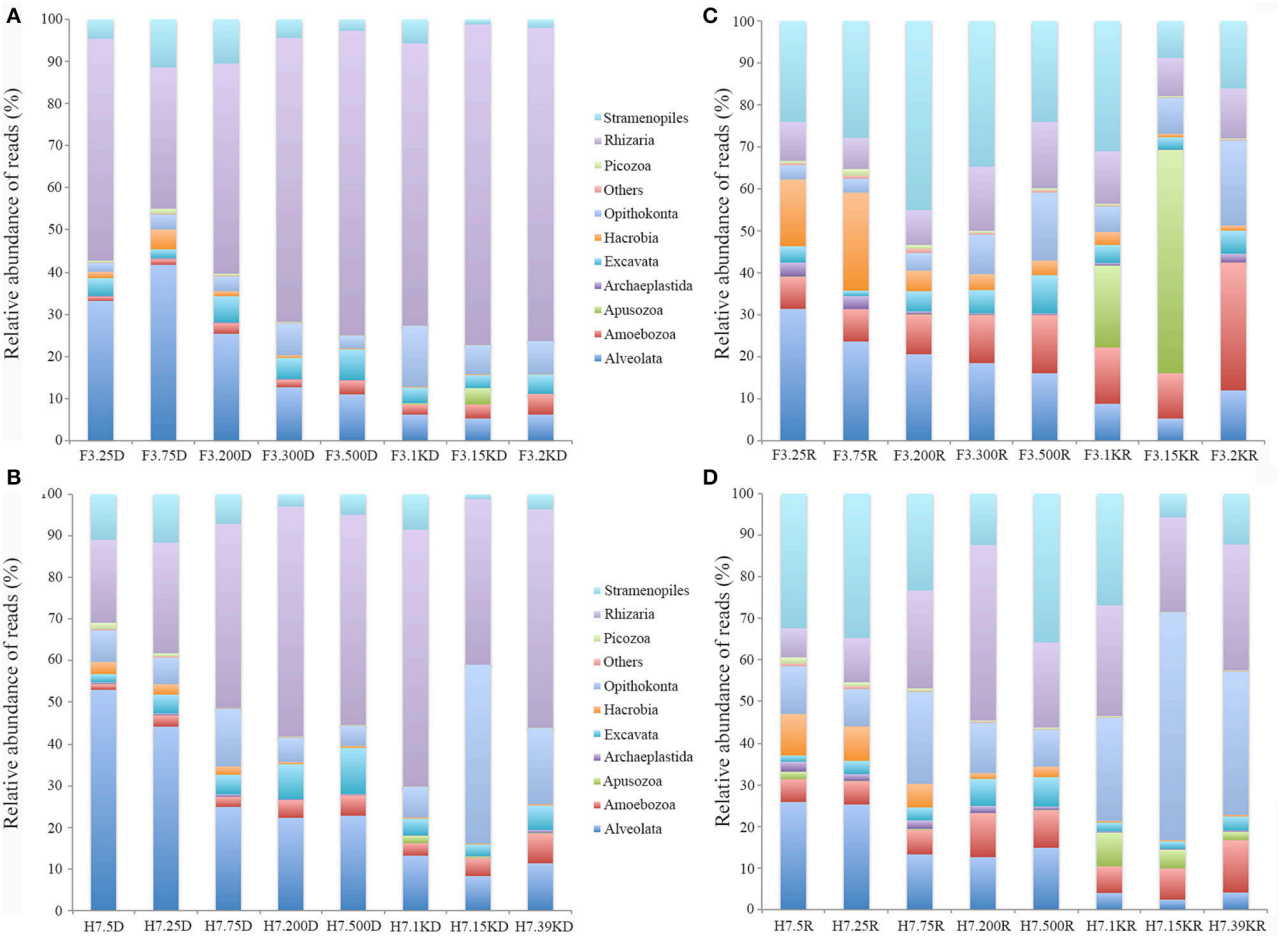
Relative sequence abundance of microbial eukaryotes in the water column at the two sampling sites, as revealed by DNA **(A,B)** and RNA **(C,D)** surveys.

Several supergroups followed clear depth-specific trends in the DNA survey (Figure 4). Alveolata usually dominated the shallow waters but its relative abundance decreased with increasing depth, ranging from 41.7 to 5.3% at site F3 and from 53.1 to 8.3% at site H7 (Figures 4A,B). The relative contribution of Rhizaria showed a reverse trend, with relative abundance generally increasing with the increase of depth, especially at site F3 where Rhizaria contributed more than 60% of all microbial eukaryote reads in waters below 300 m. The contribution of Opisthokonta generally increased with increasing depth, accounting for 14.4% of total reads at 1,000 m at site F3 and up to 43% at 1,500 m at site H7. The contribution of Stramenopiles was small across all water depths but generally higher in shallow than deep waters. The contribution of Amoebozoa was also small across all water depths but their relative abundance slightly increased with increasing depth. The other groups (Picozoa, Hacrobia, Excavata, Archaeplastida and Apusozoa) made only minor contributions, generally less than 10% of the total reads individually (Figures 4A,B).

Similar to the DNA survey, the active microbial eukaryotic assemblages as revealed by RNA also showed depth-related trends in the water column (Figures 4C,D). The contributions of Rhizaria reads were generally higher in deep than shallow waters, accounting for 7.4 to 15.8% at site F3 and 7 to 42.2% at site H7, peaking at 500 m and 200 m, respectively. The contributions of Stramenopiles reads ranged from 8.8 to 45% of the total reads at site F3, peaking at 200 m and 5.7 to 35.8% at H7 with a peak at 500 m. In contrast to these two groups, Alveolata reads generally decreased with increasing water depth, ranging from 5.3 to 41.7% at site F3 and 2.4 to 25.9% at site H7. Opisthokonta reads reached their highest contribution (up to 20.3% of total reads) at 2,000 m at site F3 and 54.7% at 1,500 m at site H7. The contribution of Amoebozoa reads did not vary much across different depths, peaking at 2,000 m at site F3 (30.5%). The other groups (Picozoa, Hacrobia, Archaeplastida, Excavata, and Apusozoa) were minor constituents of the total microbial eukaryote assemblages except at 1,500 m at site F3 where the contribution of Apusozoa was 53.2% of the total reads.

The four most consistently abundant groups within Alveolata were Ciliophora, Dinophyceae, MALV-I, and MALV-II, each showing various relative contributions to both DNA and RNA datasets (Figure S5). Reads affiliated with Dinophyceae dominated the alveolate assemblages in the DNA dataset, whereas those affiliated with Ciliophora, most of which in the euphotic zone of open oceans are active grazers of prokaryotes and pico-/nano-sized eukaryotes (Sherr and Sherr, 1987), contributed more in the RNA dataset and were the dominant alveolates at some water depths (e.g., 2,000 m at F3; 5, 25, 75, and 500 m at H7; Figure S5A). Within Stramenopiles, MAST reads dominated the shallow water and showed substantial depth-specific variation in both the DNA and RNA surveys (Figure S5B). In deep waters, Bicoecea replaced MAST as the dominant Stramenopiles group with up to 85% of total stramenopile reads. The relative contributions of photosynthetic Stramenopiles (Dictyochophyceae, Pelagophyceae, Pinguiophyceae, Chrysophyceae, Synurophyceae, and Bacillariophyta) were larger in the RNA than the DNA survey. Surprisingly, their contributions in the deep water were sometimes comparable to those in shallow waters, even in the RNA dataset (Figure S5B). Within Rhizaria, the most dominant group in both DNA and RNA surveys was Spumellarida, the contribution of which generally increased with increasing water depth (Figure S5C). Within Hacrobia, Haptophyta was the most dominant group in both DNA and RNA surveys, its contribution to total sequences being larger in the RNA than in the DNA survey (Figure S6A). Another photoautotrophic group, Cryptophyta, which was almost undetectable in DNA survey, contributed more in the RNA survey (Figure S6A). Opisthokonta was dominated by Fungi both in DNA and RNA surveys; Choanoflagellida contributed more in the RNA than the DNA survey and its contribution to total sequences was generally larger in the deep than shallow waters (Figure S6B).

### Relative activity of major groups of microbial eukaryotes

**Figure 5:**
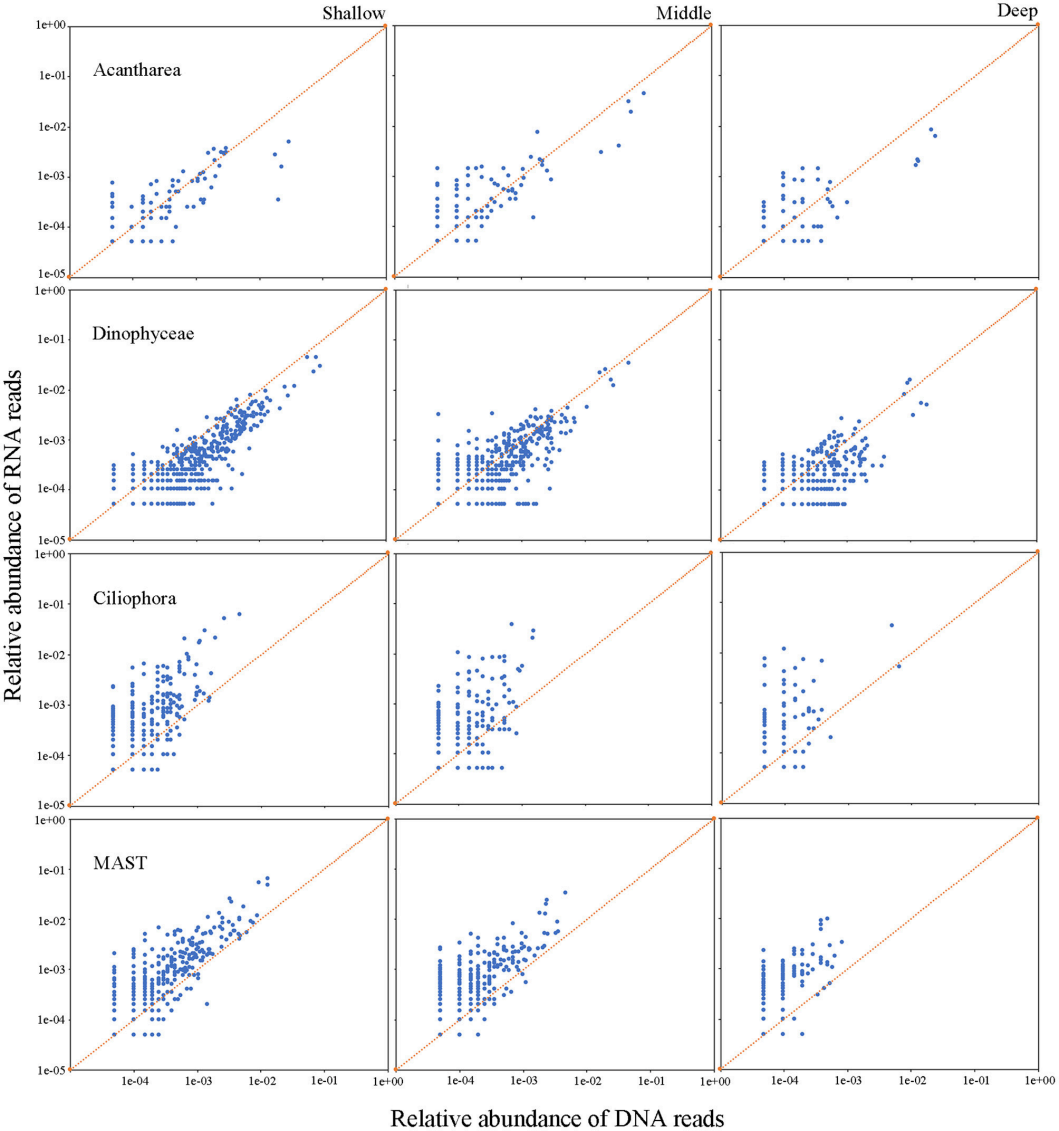
Relative abundance of RNA (y-axis) and DNA (x-axis) reads for each OTU in representative microbial eukaryotes assemblages, Acantharea, Dinophyceae, Ciliophora, and MAST, in the water column of the South China Sea.

Analyses of the relative metabolic activity for major microbial eukaryote assemblages were conducted separately by employing RNA: DNA ratio as a proxy of relative activity. This was achieved by combing samples into three groups according to the water depth from which samples were collected: shallow (5, 25, and 75 m), middle (200, 300, and 500 m), and deep (1,000, 1,500, 2,000, and 3,900 m) waters. Most but not all major groups of microbial eukaryotes showed depth-related distribution patterns for their relative activities. The activities of Cercozoa, Acantharea, and RAD-B, which are all members of Rhizaria, showed similar depth-related patterns of relative activity throughout the water column. Their activities were significantly higher in middle (average RNA: DNA ratio of 4.0, 2.4, and 1.3, respectively), followed by deep (average RNA: DNA ratios of 3.7, 2.1, and 1.2, respectively) and shallow (average RNA: DNA ratio of 2.4, 1.5, and 0.9, respectively) waters (Table 2; Figure 5, Figure S7). The activity of Polycystinea, another major group of Rhizaria, was the highest in shallow (1.4) followed by middle (0.9) and deep (0.5) waters. (Table 2; Figure S7). Ciliophora, mainly represented by pelagic groups such as Choreotrichia, Oligotrichia, and Scuticociliatia, were overrepresented in RNA surveys and showed no significant differences of activity in different depth groups (on average of 5.7, 5.7, and 8.2, respectively) (Table 2; Figure 5). Dinophyta, represented primarily by Dinophyceae, MALV-I, and MALV-II, were overrepresented in DNA surveys (Figure 5, Figures S7, S8). The activities of Dinophyceae and MALV-I were the highest in middle followed by deep and shallow waters. For MALV-II, the highest activity was found in middle followed by shallow and deep waters. Stramenopiles represented mainly by Bacillariophyta, MAST, and other photoautotrophic groups (Chrysophyceae, Synurophyceae, Dictyochophyceae, and Pelagophyceae) were overrepresented in the RNA compared to the DNA survey even in deep waters (Table 2). The highest activities of Bacillariophyta and MAST were found in deep followed by middle and shallow waters (Figure 5, Figure S8). No significant difference of activity was found in the other main photoautotrophic groups of Stramenopiles (Chrysophyceae, Synurophyceae, Dictyochophyceae, and Pelagophyceae) in the water column. For the other groups of microbial eukaryotes, Amoebozoa, Archaeplastida (mainly represented by Chlorophyta and Streptophyta), Choanoflagellatea, and Hacrobia (mainly represented by Cryptophyta, Haptophyta, and Telonemia), the average RNA: DNA ratios were above 1:1 regardless of the water depth and their activity did not show any significant difference between shallow, middle and deep water groups (Table 2). The highest activity of Fungi and Excavata (mainly represented by Discoba) was found in middle waters, followed by deep and shallow waters (Table 2; Figure S8).

**Table 2:** Average RNA: DNA ratios for major groups of microbial eukaryotes in shallow (5, 25, and 75 m), middle (200, 300, and 500 m), and deep (1,000, 1,500, 2,000, and 3,900 m) waters.

		**Average RNA: DNA ratios**			**Pairwise comparisons**		
		**Shallow**	**Middle**	**Deep**	**Shallow-Middle**	**Shallow-Deep**	**Middle-Deep**
Rhizaria	Cercozoa	2.38	4.00	3.68	**0.030**	0.215	0.495
	Acantharea	1.47	2.43	2.08	**0.017**	0.096	0.573
	Polycystinea	1.42	0.92	0.46	**0.044**	**0.001**	0.066
	RAD-B	0.87	1.26	1.15	**0.036**	0.156	0.539
Alveolata	Ciliophora	5.67	5.74	8.17	0.944	0.075	0.089
	Dinophyceae	0.68	1.33	0.93	**0.000**	0.077	**0.006**
	MALV-I	0.47	0.84	0.71	**0.000**	**0.012**	0.180
	MALV-II	0.79	0.92	0.74	0.056	0.575	**0.037**
Stramenopiles	Bacillariophyta	2.44	5.62	8.93	**0.000**	**0.000**	**0.005**
	Photosynthetic Stramenopiles^*^	5.07	5.58	5.52	0.555	0.419	0.807
	MAST	3.94	5.26	5.91	**0.014**	**0.001**	0.252
Amoebozoa		7.02	4.92	5.33	0.208	0.285	0.799
Archaeplastida		7.91	9.17	3.98	0.341	0.545	0.138
Opisthokonta	Choanoflagellatea	5.20	9.34	8.91	0.065	0.174	0.881
	Fungi	1.37	3.28	2.76	**0.004**	**0.035**	0.372
Excavata		1.02	1.43	1.27	**0.002**	0.069	0.180
Hacrobia		5.41	7.00	5.79	0.092	0.738	0.333

*Difference in RNA: DNA ratios between shallow, middle, and deep waters were evaluated by analysis of variance (ANOVA) for significantly difference (P < 0.05). Bolded values are significant*.

* *Phototrophic Stramenopiles included Chrysophyceae, Synurophyceae, Dictyochophyceae, and Pelagophyceae*.

## Discussion

### Analysis of RNA reduces the interference of metazoan sequences in protistan surveys

In the present study, metazoan sequences were often encountered, especially in the DNA dataset, even after pre-filtration using different sizes of mesh. The presence of metazoan sequences probably came from extracellular metazoan DNA after death or leakage from damaged metazoans during the filtration process. In the shallow waters (5, 25, 75 m), their contributions were over 10% in most samples. In 5 m and 25 m waters at site H7, metazoan sequences accounted for up to 41.7 and 32.7% of the total eukaryote sequences, respectively. In the RNA dataset, however, the contributions of metazoan sequences were notably smaller in most samples, the only exception being 25 m depth at site H7 in which the contribution of metazoan sequences was higher in the RNA than in the DNA dataset. In the middle and deep water samples, the contributions of metazoan sequences were comparably smaller than in the shallow waters both in DNA and RNA surveys. Although the contributions of metazoan sequences revealed by DNA did not differ as dramatically as those revealed by RNA in middle and deep waters, they still contributed less in the RNA than in the DNA surveys. A recent study that analyzed samples collected seasonally at several sites in the eastern North Pacific also found that the contributions of metazoan sequences were lower in RNA than DNA surveys (Hu et al., 2016). Thus, the application of RNA sequencing can serve as an alternative approach to reduce the interference of metazoan sequences in protistan surveys even for deep water samples.

### Alpha and beta diversity of microbial eukaryotes in the bathyal zone of the South China sea

Several studies on the diversity of microbial eukaryotes in the water column have been carried out in the world's oceans and depth has been identified to be one of the major environmental shaping factors (Countway et al., 2007; Schnetzer et al., 2011; Xu et al., 2017). In the present study, depth-related changes in the protist community structure based on the DNA dataset were consistent with those based on the RNA dataset (Figures 2, 4, Figure S4). Therefore, the “total” (as seen in DNA dataset) and the “active” (as seen in RNA dataset) communities of microbial eukaryote were both shaped by water depth. Several studies based on analyses of DNA from samples collected at local or regional scales have shown Rhizaria to be the dominant micoeukaryote group in the deep sea (Countway et al., 2007; Not et al., 2007; Orsi et al., 2011; Schnetzer et al., 2011; Xu et al., 2017). Recently, a large-scale survey on the diversity of bathypelagic microbial eukaryotes also revealed Rhizaria being a dominant group in the world's oceans (Pernice et al., 2015). However, whether Rhizaria is the dominant functional group in deep ocean water was still unclear as most previous studies are based on analyses of DNA. Compared with DNA, the RNA molecule is much less stable and consequently has much a shorter lifetime outside the cell (Karl and Bailiff, 1989). Thus, RNA can serve as an indicator of metabolic activity in microbial communities (Stoeck et al., 2007). Nevertheless, few studies have been performed that include simultaneous DNA and RNA analyses from the same sample source (Logares et al., 2014; Massana et al., 2015). Not et al. (2009) compared the community composition of pico-sized microeukaryotes in the euphotic zone of the Mediterranean Sea based on both DNA and RNA data and found that the contribution of radiolarian sequences, which overwhelmingly dominated DNA libraries, was significantly less in the RNA libraries. In the present study, the proportion of Rhizaria-affiliated reads in deep layers reached up to ca. 75% in the DNA dataset. However, in the RNA extracts, the proportion of rhizarian reads was significantly lower throughout the water column (Figure S9), indicating that some of the deep rhizarian sequences may come from dead or dormant, rather than metabolically active, cells. However, the RNA approach did reveal the presence of Radiolaria sequences in the deep sea and a depth-related distribution pattern. Previous studies have shown the presence of new radiolarian clades in the deep sea (Orsi et al., 2011; Xu et al., 2017). Our study provides evidence for the presence of active assemblages of radiolarians in the deep sea, so these new clades might represent active radiolarian assemblages.

In the cluster analysis, samples were first clustered into two groups by nucleic acid source, i.e., the RNA group and the DNA group (ANOSIM, R = 0.5506, *p* = 0.0001). Within each group, the samples generally clustered into subgroups by water depth (Figure 2). A previous study on benthic prokaryotes from an active mud volcano in the Gulf of Mexico also found that the communities revealed by DNA differed significantly from those revealed by RNA and this difference was more significant than that caused by the sediment depth (Martinez et al., 2006). Lanzén et al. (2011) explored prokaryotes at the Jan Mayen hydrothermal vent field and found that the composition and diversity predictions differed systematically between extracted DNA and RNA samples. An investigation of bacterial communities in Antarctic coastal waters using both 16S RNA and 16S DNA showed similar results (Gentile et al., 2006). In the present study, however, although DNA and RNA showed a distinctly different community composition for the same sample, the two approaches revealed similar or comparable community structure (depth-related gradients) in each sample. Combined with the above evidence, our results support the notion that nucleic acid source has a greater influence than depth (as seen in the present study), or other environmental factors (as seen in the above references), in determining eukaryote microbial community composition. The differences of community composition revealed by DNA and RNA were likely caused by the differences between the activity levels of taxa because the former generally reflects activity and the latter usually includes active, dormant, and dead cells (Lanzén et al., 2011). In addition, the techniques applied, e.g., the bias introduced during the extraction of DNA/RNA and reverse transcription, may also contribute to these differences. Thus, environmental RNA sequencing can serve as a reliable approach to reveal the diversity and community structure of active microorganisms and provide supplementary perspectives of microbial eukaryotes. Care should be taken, however, when comparing communities, especially if data is derived from different nucleic acid sources, i.e., DNA and RNA.

### Metabolic activities of major microbial eukaryotic groups in the bathyal zone of the South China sea

Comparisons of RNA and DNA sequence abundance (RNA: DNA ratios) have been widely used as proxies of *in situ* microbial metabolic activity (Poulsen et al., 1993; Logares et al., 2014; Massana et al., 2015). However, this approach has only recently been applied to the study of microbial eukaryotes (Charvet et al., 2014; Massana et al., 2015; Hu et al., 2016). Each of these studies focused on microbial eukaryote communities either above the euphotic/disphotic zones or in shallow coastal water sediments; the metabolic activity of microeukaryotes in deep sea (>1,000 m) pelagic waters remains largely unknown. Therefore, to our best knowledge, the present study is the first investigation of the relative metabolic activity of microbial eukaryotes in the water column from the surface to bathypelagic depths (>3,500 m). RNA-DNA comparisons of the major components of the microeukaryote communities in the water column of the South China Sea revealed depth-related pattern for their relative activity: the highest activity of most groups (e.g., Cercozoa, Acantharea, RAD-B, Dinophyceae, MALV-I, MALV-II, Fungi, and Excavata) was found in middle waters. The highest activity of Bacillariophyta and MAST was found in deep waters and that of Polycystinea was found in shallow waters. Unexpectedly, however, we did not, find any depth-related trends in the relative activity of some heterotrophic groups, e.g., Ciliophora, Amoebozoa, Choanoflagellatea, and even some photoautotrophic groups such as Stramenopiles (represented by Chrysophyceae, Synurophyceae, Dictyochophyceae, and Pelagophyceae), Archaeplastida, and Hacrobia, and their relative activities did not change significantly along depth gradients.

Ciliates have long been known as the major grazers of prokaryotes and pico-/nano-sized eukaryotes in the euphotic zone (Sherr and Sherr, 1987). However, data on the abundance and community structure of ciliates in the deep sea are scarce compared with prokaryotes (Nagata et al., 2010). The roles of ciliates in deep-sea microbial food webs thus remain enigmatic, largely because of the lack of metabolic data (Nagata et al., 2010). One study that surveyed protistan assemblages along European coasts showed ciliates were overrepresented in RNA compared to DNA datasets, both in pico- and nano-sized fractions of waters and sediments (Massana et al., 2015). A study at a coastal ocean site in the eastern North Pacific showed higher ciliate activity in April compared to other months and higher activity below the euphotic zone compared to shallower depths (Hu et al., 2016). In the present study, relative metabolic activity of deep-sea ciliates was comparable to that of those in the euphotic zone (Figure 5), which indicates: (1) the presence of active ciliates in the bathyal zone of the South China Sea, and; (2) ciliate activity did not decrease with increasing depth. Previous studies showed that ciliate abundance varies in the range of <0.1–52 cells L^−1^ in deep (>1,000 m) waters (Nagata et al., 2010 and references therein). A parallel study on ciliates in the water column at the same two sites in the South China Sea using quantative protargol staining (QPS) method showed that their abundances were <1–7 cells L^−1^ in the waters below 1,000 m depth (data not shown), which is similar to previous studies (Nagata et al., 2010 and references therein). Although the abundance of ciliates in the deep sea is much lower than prokaryotes and heterotrophic nanoflagellates, the discovery of high ciliate metabolic activity in the bathyal zone of the South China Sea indicates that they are probably grazing on such prey and are thus likely playing an important role in controlling biogeochemical processes in the deep sea. Unfortunately, the present study did not employ time-series sampling so temporal variations in the activity of deep-sea ciliates remain unknown.

Rhizaria and Dinophyta were predominant in the DNA dataset and their RNA: DNA ratios were on average below 1:1. (Table 2; Figure 5, Figure S7). The highest activity of Dinophyceae and MALV-I were found in middle water followed by deep and shallow waters in the present study. The highest activity of MALV-II was also found in middle water but followed by shallow then deep waters. The recent San Pedro Ocean Time-series (SPOT) study of protist diversity and activity at a coastal site in the eastern North Pacific showed high RNA: DNA ratios for Radiolaria at the subsurface chlorophyll maximum layer, 150 and 890 m (Hu et al., 2016). Similarly, generally high RNA: DNA ratios for dinoflagellates were found in all euphotic zone samples in April (Hu et al., 2016). By contrast, Massana et al. (2015) reported that: (1) most lineages of Radiolaria were equally well represented in DNA and RNA surveys of pico-, nano-, micro/meso-sized plankton and sediment samples collected from European coastal waters; (2) Acantharea was overrepresented in RNA surveys of the micro/meso-sized plankton; and (3) RAD-B was overrepresented in DNA surveys of sediments. Therefore, it seems likely that the activities of Radiolaria and Dinophyta might be influenced by geographical location, water depth, and season. In the present study, another major lineage of microeukaryotes, Stramenopiles, also showed high RNA: DNA ratios in the water column (Table 2), which is consistent with the findings of Massana et al. (2015).

Overall, we did find depth-related distribution patterns for most of the major groups of microbial eukaryotes. Surprisingly, the highest activity for most groups was usually found in middle (200, 300, and 500 m) rather than shallow (5, 25, and 75 m) or deep (1,000, 1,500, 2,000, and 3,900 m) waters. More than 90% of the organic carbon annually exported from surface waters is respired back to CO_2_ in the mesopelagic waters (generally the depth range from 200 to 1,000 m) by prokaryotes (Arístegui et al., 2009). Although still being controversial, grazing of prokaryotes by small eukaryotes such as small heterotrophic flagellates (HF) are proposed to remove most of the prokaryotic production in the mesopelagic waters (Arístegui et al., 2009). HF is a phylogenetically diverse group and include members from a variety of microbial eukaryotes such as Stramenopiles and Rhizaria. The high metabolic activity of some groups of microbial eukaryotes, such as Cercozoa, Acantharea, and Excavata, observed in the present study may help explain the active grazing impact of HF on prokaryotes in the mesopelagic waters (Arístegui et al., 2009). However, the present study did not include temporal sampling and the two water columns are represent only a tiny fraction of the global oceans. A strategy that integrates both temporal and large-scale spatial sampling is therefore needed in order to shed more light on the functions of microbial eukaryotes in marine biogeochemical cycling and their responses to environmental change.

### The presence of photoautotrophic microbial eukaryotes in the dark ocean

Previous studies based on direct microscopy have reported the presence of well-preserved phytoplankton cells in the deep sea (Kimball et al., 1963; Smayda, 1971; Wiebe et al., 1974). Healthy photoautotrophic cells, especially diatoms, were recently reported down to 4,000 m in a global survey of the dark ocean (Agusti et al., 2015). A parallel study on the diversity of bathypelagic microbial eukaryotes from the same circumnavigation expedition using pyrosequencing also showed the presence of photoautotrophic groups in the deep sea, e.g. Bacillariophyta, Boliodphyta, Dictyochophyta, Prasinophyceae, Prymnesiophyceae, Raphidophyta, and Eustigmatophyceae, which represented 0.14% of the pyrotaps and 2% of the OTUs in the sequence analyses (Pernice et al., 2015). This work was generated based on DNA sources therefore it remains unclear whether the photosynthetic signals were from live cells, dead cells, dormant cells or extracellular DNA. The DNA survey in the present study likewise recovered photoautotrophic groups which represented ca. 0.1–0.4% of the total microbial eukaryote reads in the samples ≥1,000 m. Furthermore, the RNA survey revealed that photoautotrophic groups accounted for ca. 0.9–4.3% of the total reads which is even higher than their contribution in the DNA dataset. The photoautotrophic groups were still present even after applying much stricter screening processes, i.e., considering only the OTUs represented in both RNA and DNA datasets (Figure S8). Furthermore, contrary to expectations, we did not find any decrease in the metabolic activity of some photoautotrophic groups (photoautotrophic Stramenopiles such as Chrysophyceae, Synurophyceae, Dictyochophyceae, Pelagophyceae, and members of Archaeplastida and Hacrobia) with increasing depth (Figure S8). Thus, the present study confirmed the presence of not only live but also active photoautotrophic microbial eukaryotes in the deep sea. Agusti et al. (2015) hypothesized that fast-sinking is the main reason for the presence of photoautotrophic cells in the dark ocean and that when these cells die they serve as fresh organic carbon input fueling the deep-sea ecosystem. If fast-sinking is the reason for the presence of photoautotrophic cells in the deep sea, their sinking rates must either be much faster than expected (some of these cells were within the pico-sized fraction), or they can survive in the dark for much longer than previously realized. Furthermore, we cannot rule out the possibility that some of these photoautotrophic microbial eukaryotes are facultative heterotrophs because mixotrophy in protists is well documented and such forms are thought to contribute significantly to biogeochemical cycles in the oceans globally (Mitra et al., 2014; Worden et al., 2015; Stoecker et al., 2016). If the latter is the case, there are at least two possibilities that need to be investigated in further studies: (1) that these cells come from the surface layers sink to the deep sea and survive by phagotrophy (e.g., grazing on prokaryotes), or; (2) they are endemic in the deep sea and act as grazers in the microbial loop.

## Author contributions

DX and PS conceived and designed the experiments; DX, PS, CH, RL, NJ, and AW performed onboard ample processing and analyzed data; DX, PS, AW, NJ, RL, and CH wrote the paper.

### Conflict of interest statement

The authors declare that the research was conducted in the absence of any commercial or financial relationships that could be construed as a potential conflict of interest.
