# Supplementary material for: Microbial Eukaryote Diversity and Activity in the Water Column of the South China Sea Based on DNA and RNA High Throughput Sequencing
Source: Front Microbiol. 2017 Jun 14;8:1121. doi: 10.3389/fmicb.2017.01121 (PMC5469884; doi:10.3389/fmicb.2017.01121)

FIGURE S1 Geographic locations of the sampling sites. The sampling map was generated using Ocean Data View 4 software (Schlitzer 2011).

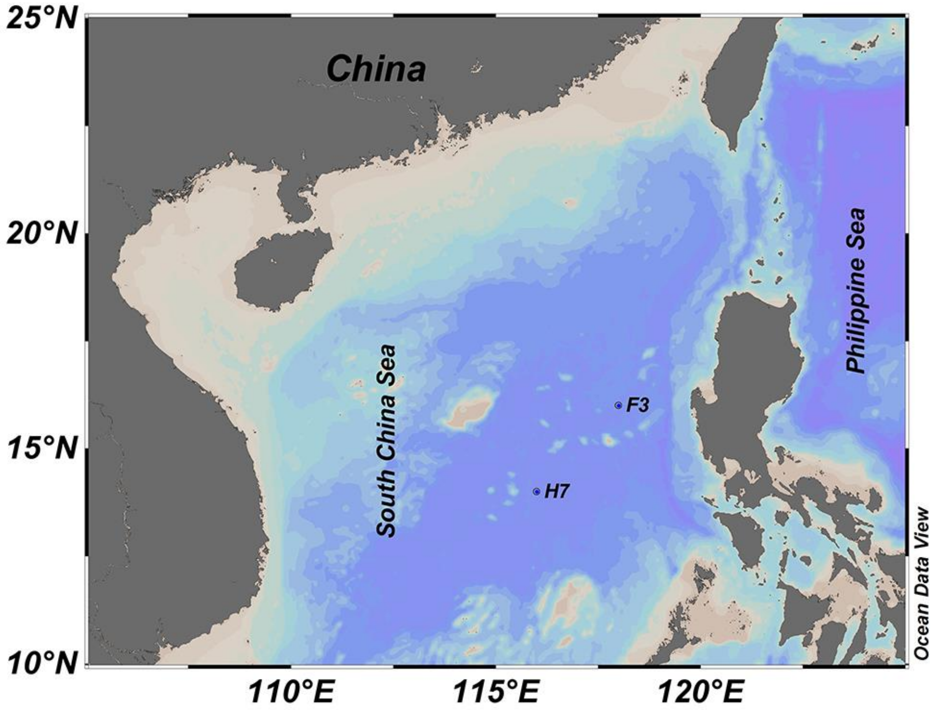

FIGURE S2 Temperature and salinity throughout the two water columns at in the water column at two sites in the South China Sea.

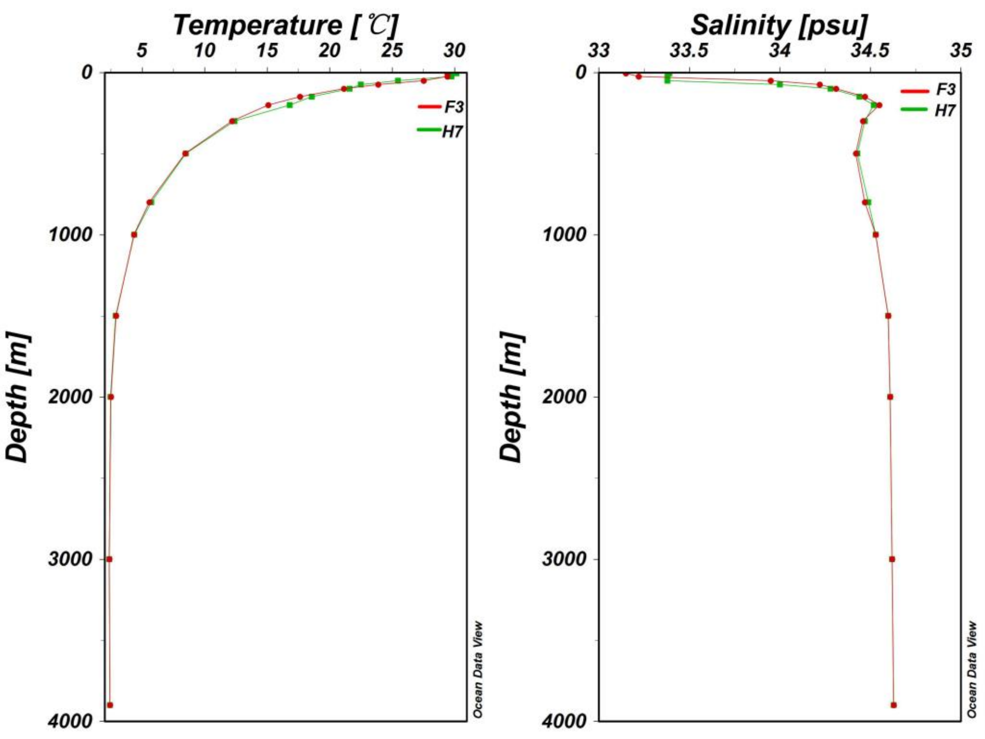

FIGURE S3 Percentages of OTU richness of major microbial eukaryotes at the supergroup taxonomic level revealed by DNA (A, B) and RNA (C, D) extracts at the two water columns, respectively.

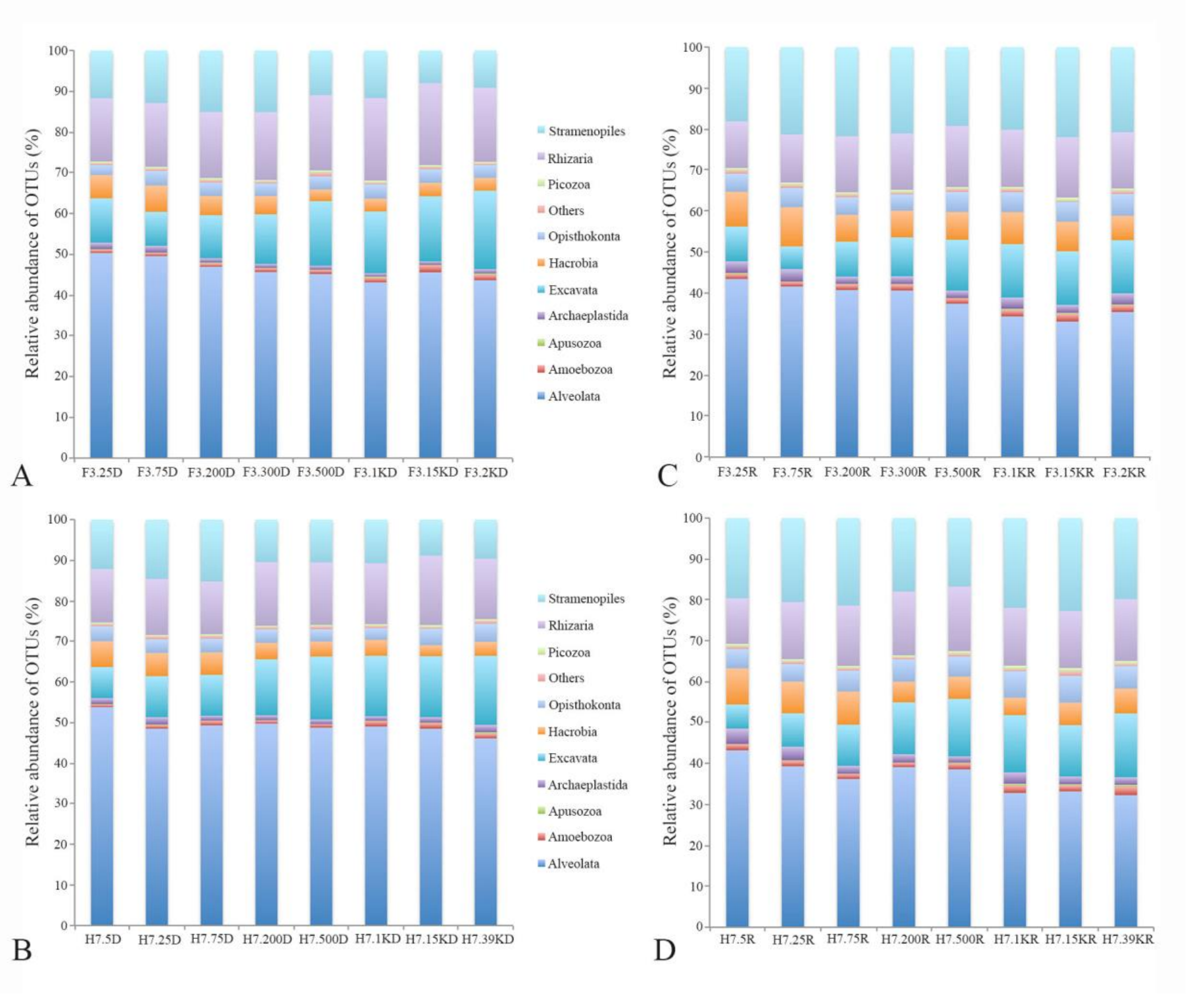

FIGURE S4 Unweighted UniFrac principal coordinates analysis (PCoA) plot of microbial eukaryotes from two water columns in the South China Sea revealed by DNA and RNA surveys.

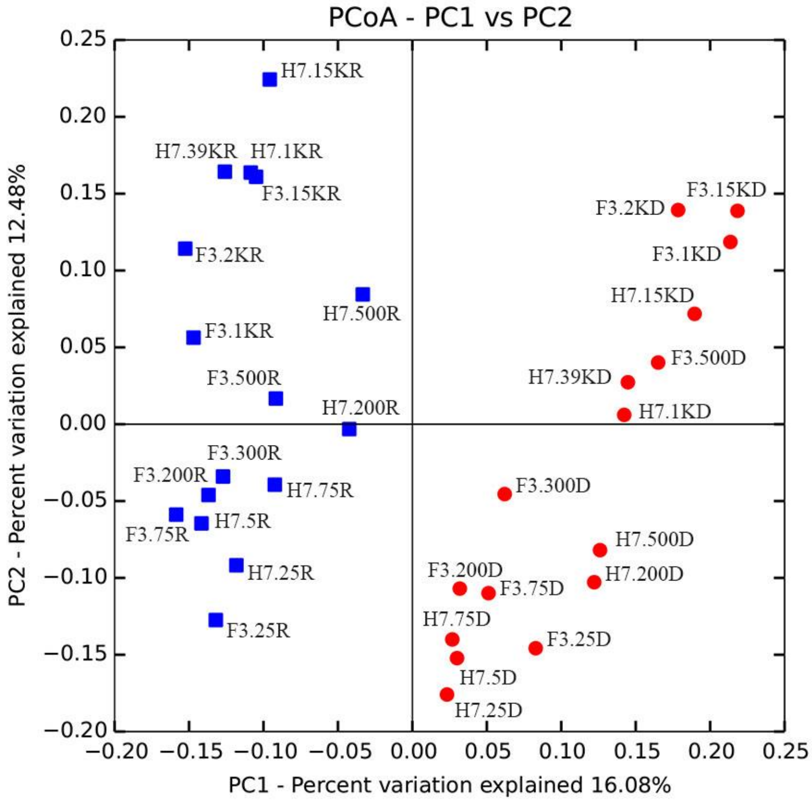

FIGURE S5 Percentages of reads of taxonomic groups in Alveolata (A), Stramenopiles (B), and Rhizaria (C) in the two water columns revealed by DNA and RNA surveys.

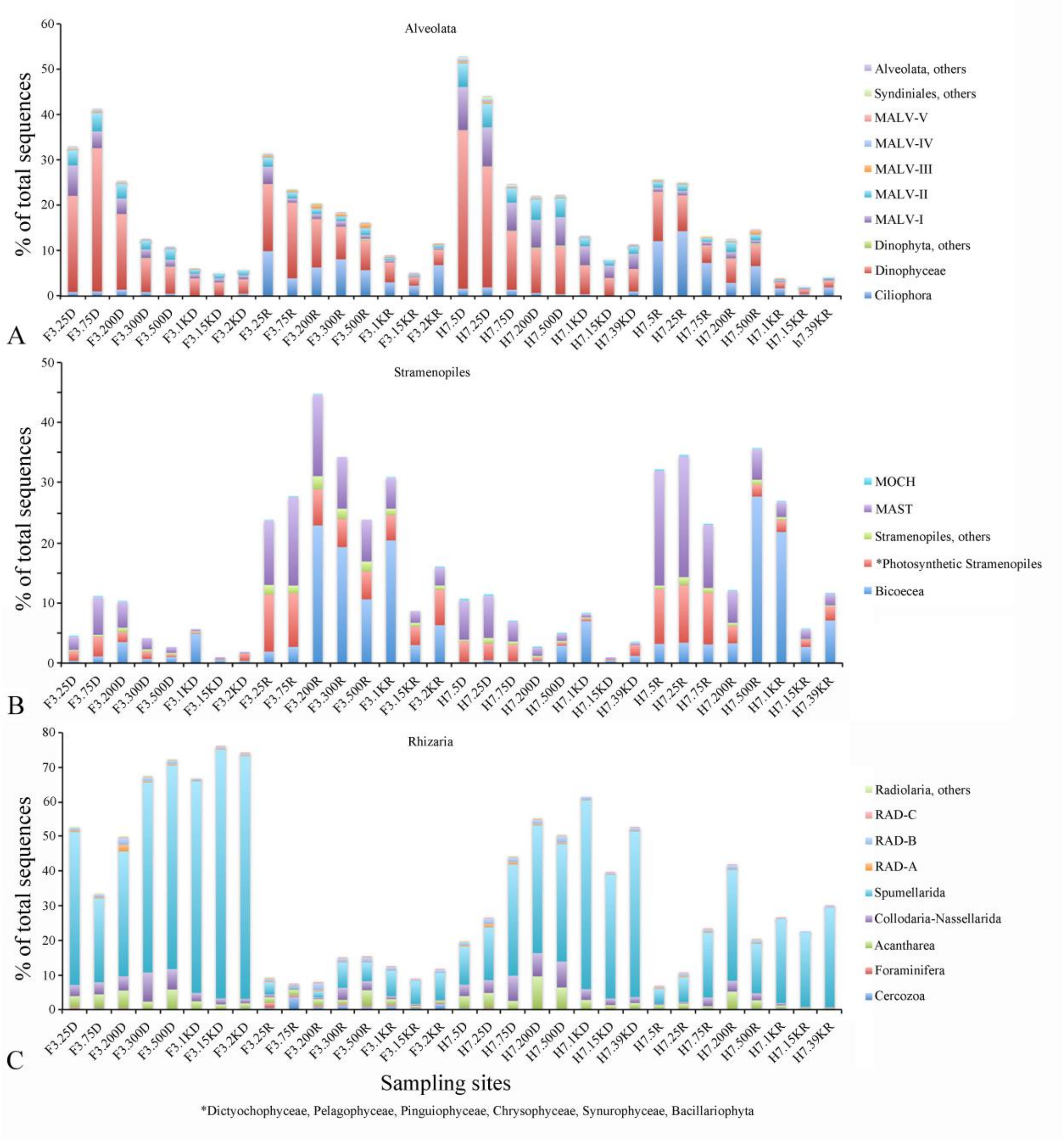

FIGURE S6 Percentages of reads of taxonomic groups in Hacrobia (A) and Opisthokonta in the two water columns revealed by DNA and RNA surveys.

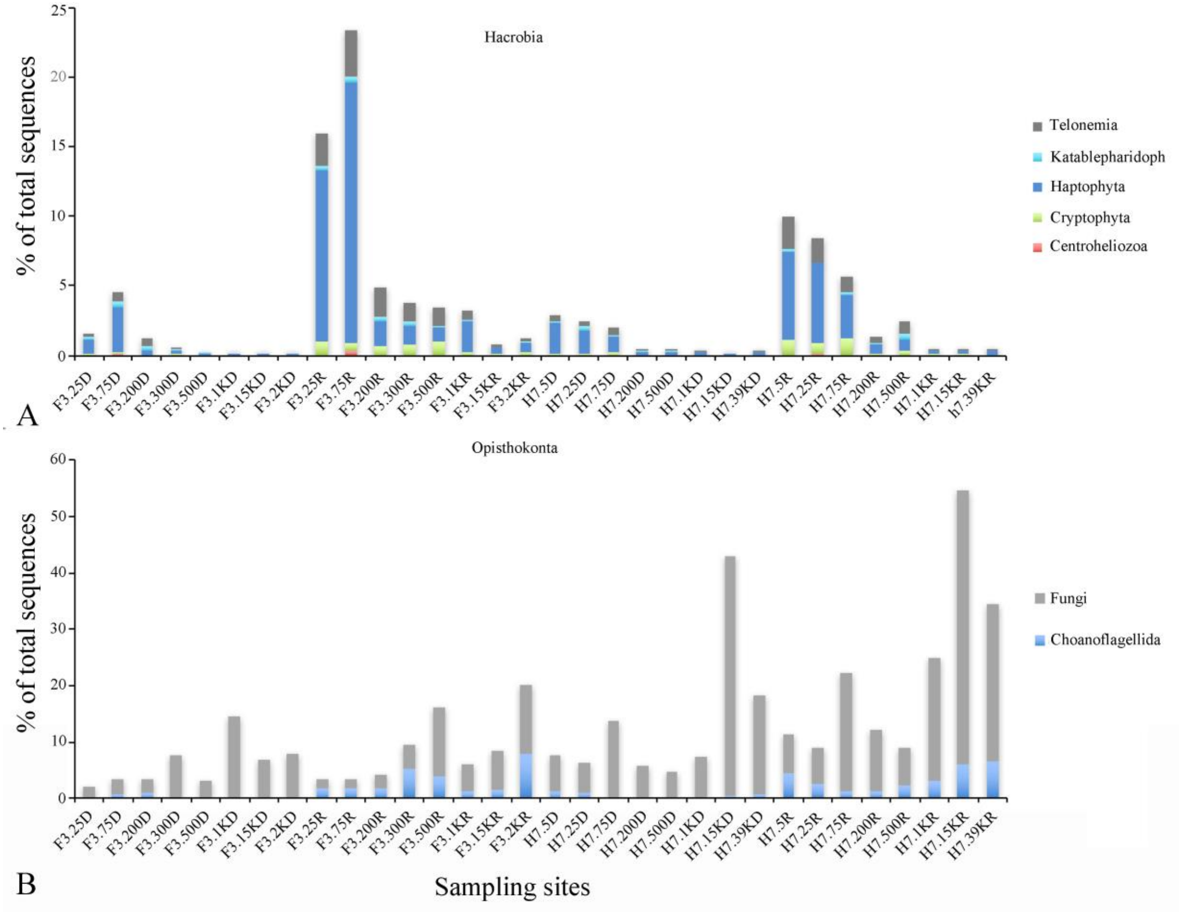

FIGURE S7 Relative abundance of RNA (y-axis) and DNA (x-axis) reads for each OTU in representative microbial eukaryotes assemblages, Cercozoa, Polycystinea, RAD-B, and MALV-I, in the water column of the South China Sea.

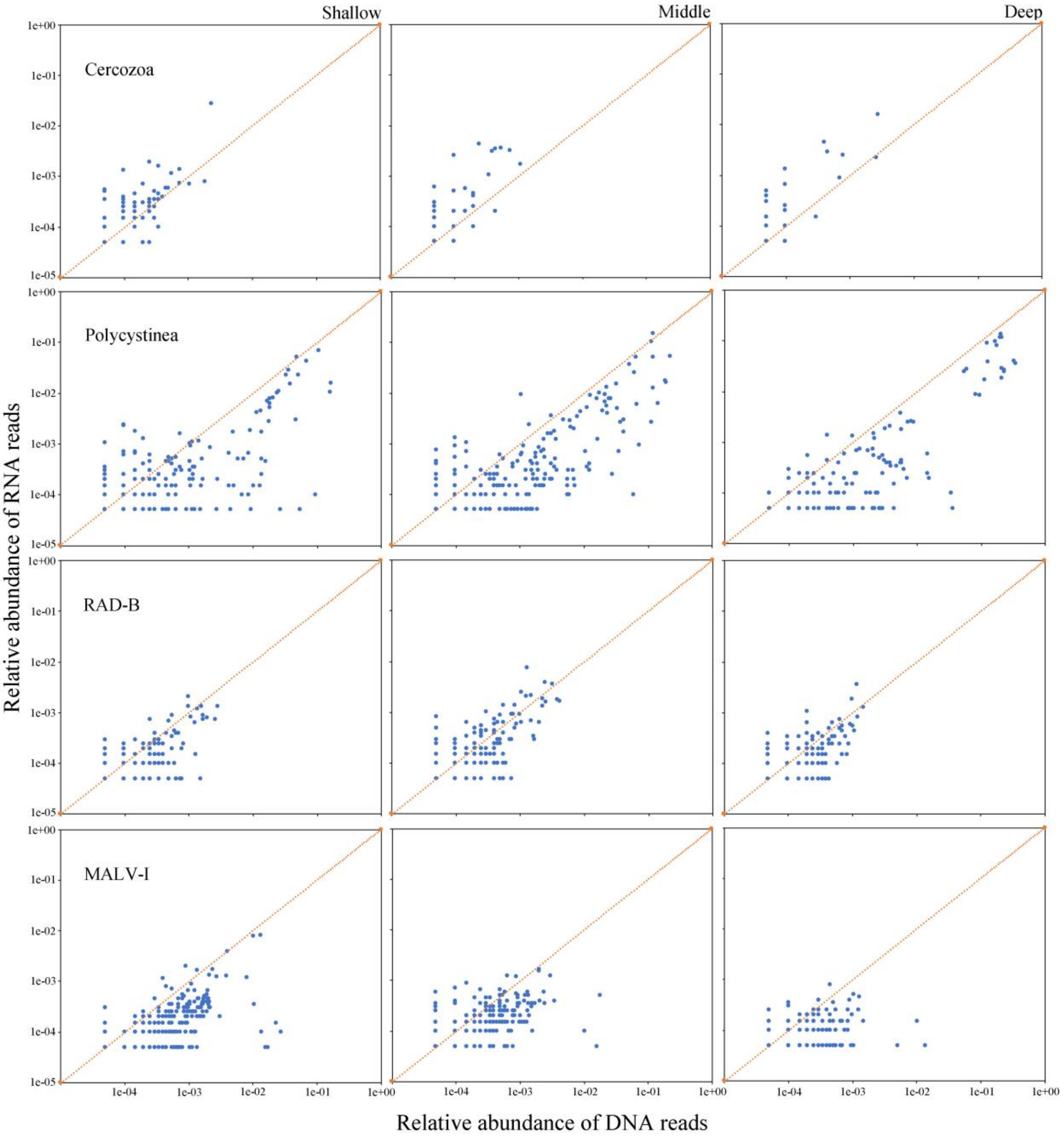

FIGURE S8 Relative abundance of RNA (y-axis) and DNA (x-axis) reads for each OTU in representative microbial eukaryotes assemblages, MALV-II, Bacillariophyta, Phototrophic Stramenopiles (represented by Chrysophyceae, Synurophyceae, Dictyochophyceae, and Pelagophyceae), and Excavata, in the water column of the South China Sea.

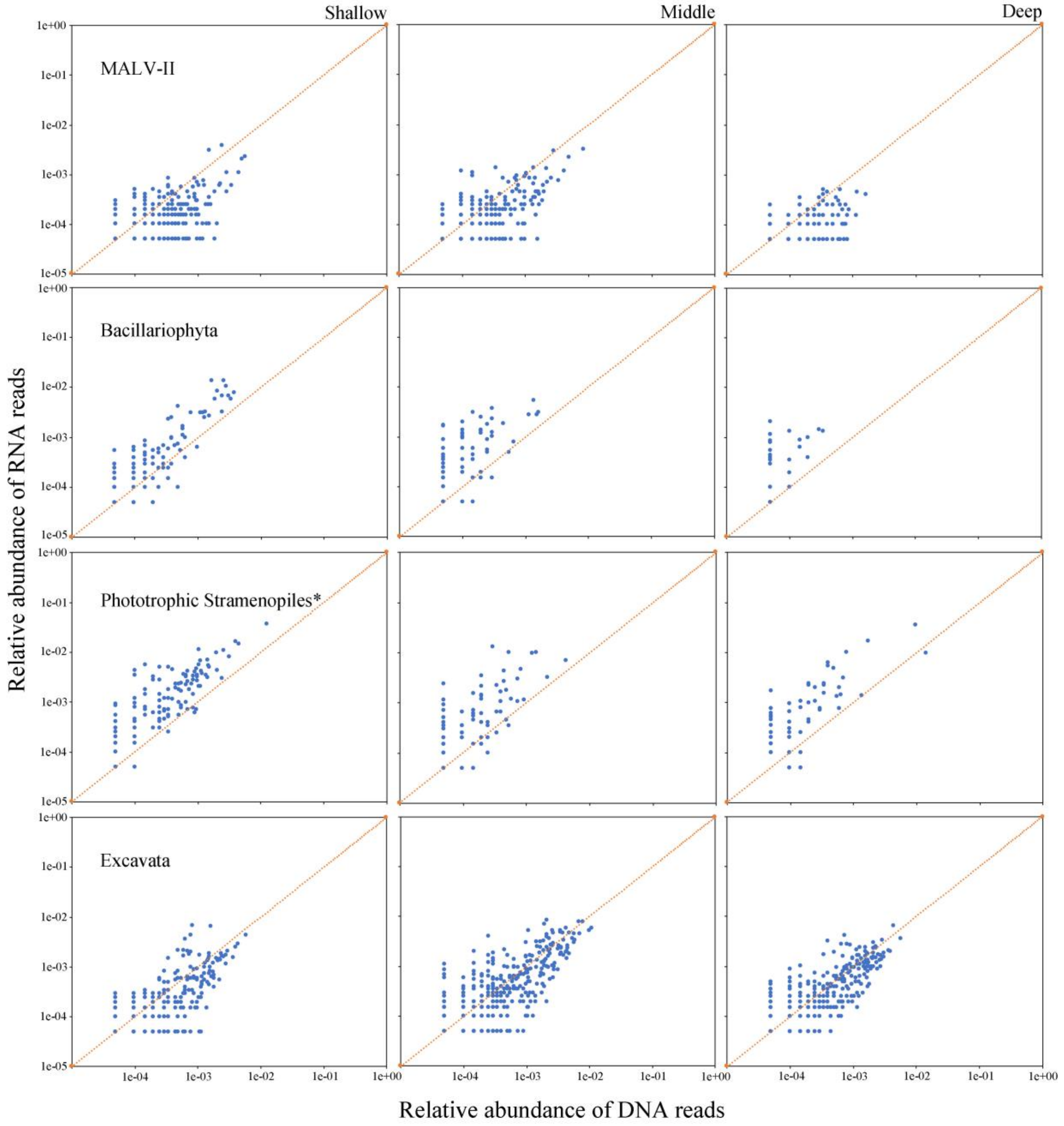

FIGURE S9 Comparisons of relative abundance of major microbial eukaryotes assemblages in DNA and RNA surveys pooled by water depths (shallow, middle, and deep). Asterisks indicate significant difference ($p < 0.05$) between DNA and RNA surveys.

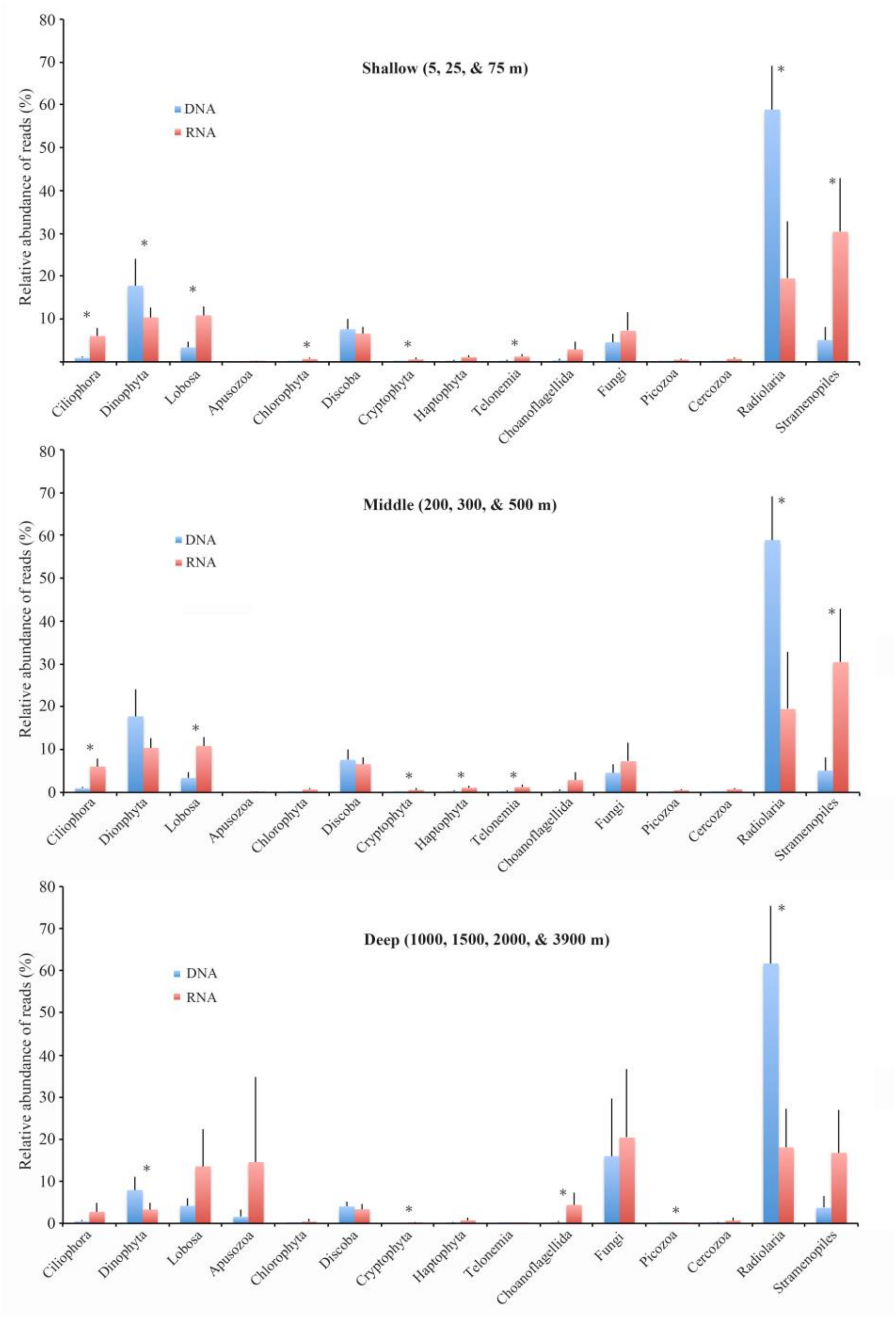

Supplement: Supplementary file 1 [file Image1.PDF]
